# Differential Metabolic Effects of Novel Cilostamide Analogs, Methyl Carbostiryl Derivatives, on Mouse and Hyperglycemic Rat

**Published:** 2012

**Authors:** Azar Hosseini, Reza Shafiee-Nick, Nasser Pour Ali Behzad, Hamid Sadeghian

**Affiliations:** 1 *Pharmacological Research Centre of Medicinal Plants, Department of Pharmacology, Faculty of Medicine, Mashhad University of Medical Sciences, Mashhad, Iran*

**Keywords:** Cilostamide, Glycogen, IBMX (3-isobutyl-1-methyl xanthine), Insulin, Milrinone

## Abstract

**Objective(s):**PDE3 has a functional role in insulin secretion and action. We investigated the metabolic effects of new synthetic PDE3 inhibitors (mc1, mc2, mc5 and mc6), on mice and hyperglycemic rat.

**Materials and Methods:**The test compound or solvent was injected subcutaneously to mice, for 7 days. On day 8, blood and liver samples were obtained. In hyperglycemic rat, 0.5 g/kg glucose with or without test compounds was injected, and followed with infusion of 1.5 g/kg/hr glucose. Blood samples were collected in mentioned intervals and liver was dissected.

**Results:**In hyperglycemic rat, all test compounds decreased blood glucose and the effect of milrinone was potentiated by glybenclamide. Milrinone or IBMX did not change plasma insulin levels, but it was augmented by combination of milrinone and glybenclamide. In both species, liver glycogen storage was decreased by IBMX, mc5, mc6 or MCPIP, increased by mc2 (liver glycogen, rat, control=56±2, mc2=70±3 *P*< 0.01, mice, control=33±0.7, mc2=42±2.3 *P*< 0.01) and was not changed in the presence of mc1. Milrinone did not change the glycogen storage in rats though increased it in mice (control= 33±0.7, milrinone= 40±1 *P*< 0.05).

**Conclusion:**Increasing plasma insulin levels by combination of milrinone and glybenclamide confirmed that in hyperglycemic rat, the hypoglycemic effect was correlated with increasing insulin secretion. Variations of plasma insulin were obscured by the pulsative characteristic of pancreatic insulin release. Decreasing glycogen storage reflected inhibition of liver PDE activity. The reasons for ineffectiveness of mc1, anabolic effect of mc2, and differential effects of milrinone were not clear.

## Introduction

Cyclic AMP (cAMP) plays a critical role in the function of pancreatic islets, hepatocytes and adipocytes (1, 2). In pancreatic islets, increasing the level of cAMP potentiates glucose-induced insulin secretion (GIIS). Gut incretins such as glucagon-like peptide-1 (GLP-1) and glucose-dependent insulinotropic peptide (GIP) mediate their potentiating effects on GIIS mainly by increasing beta cell cAMP (1, 3). In hepatocytes and adipocytes, cAMP mediates the effects of glucagon and other physiological insulin antagonists thereby glycogenolysis and lipolysis are increased via elevating cAMP levels (4). 

The intracellular level of cAMP is regulated by the rate of synthesis and degradation (3). Activators of adenylyl cyclase (AC) and inhibitors of cyclic nucleotide phosphodiesterases (PDEs) augment cAMP-dependent signaling and produce a synergistic effect in combination (5). In pancreatic beta-cells AC activators, such as forskolin and non-selective PDE inhibitor such as3- isobutyl-1-methyl xanthines (IBMX) potentiate insulin release and in hepatocytes and adipocytes, they increase glycogenolysis and lipolysis (3).

Eleven PDE families (PDE1-11) have been identified according to their substrate affinities, biochemical and physical properties, mechanisms whereby they are regulated, and different sensitivities to inhibitors (4). Several studies have shown that beta cells, hepatocytes and adipocytes contain PDE1, PDE2, PDE3 and PDE4, but PDE3 is the most important concerning the regulation of insulin secretion, glycogenesis and lipogenesis (6). PDE3 family is composed of two gens, PDE3A and PDE3B. PDE3B subunit is expressed in the adipocytes, hepatocytes and pancreatic beta-cells (1). The adipocyte and hepatocytes PDE3B has a key role in the antilipolytic and antiglycogenolytic effects of insulin. Moreover, PDE3B plays a role in tissue glucose uptake by insulin. As a result, using selective PDE3 inhibitors can disturb insulin actions in spite of increasing its secretion; for example, PC3911 (selective PDE3 inhibitor) inhibited insulin-induced glucose uptake and lipogenesis while it increased the insulin release (4). Also, it has been shown that in alert rats, milrinone, a selective PDE3 inhibitor, increased plasma insulin levels but inhibited insulin effect which result in increasing lipolysis and glycogenolysis (7). Furthermore, in fasted mouse, milrinone increased the levels of serum glucose (8). 

In a recent study, we investigated cardiotonic effects of methyl carbostiryl derivatives, analogs of cilostamide, on isolated rat atria. It was found that all of these compounds have inotropic properties but with different potencies. Among these compounds, MCPIP produced the highest inotropic effect comparable with IBMX. Surprisingly, the increasing inotropic effect of this compound did not accompany with increasing the rate of contraction (9). Considering the potential hyperglycemic and hyperlipidemic effects of selective PDE3 inhibitors (4), the aim of this research was to discover possible metabolic variation among these compounds which may explain the possible mechanisms for their differential cardiac effect. We investigated the chronic and acute metabolic effects of several new synthetized PDE3 inhibitors mc1, mc2, mc5 and mc6) (Table 1) in comparison with IBMX and milrinone in mouse and hyperglycemic rat respectively. 

## Materials and Methods

The test compounds were synthesized according to the procedure reported by Sadeghian *et al* (10). Milrinone and glybenclamide were purchased from Sigma Chemical Co. 3-isobutyl-1-methylxanthin (IBMX) and DMSO were provided by Fluka Chemical Co. Thiopental was supplied by Sandoz GmbH, Heparin 25000 units was provided by Rotex medica and Glucose Assay Kit (GOD-PAP method), Zeist Chem. Co. Insulin Assay Kit DiaSorin, Insik 5 or DSL-1600.

***Methods***

***In vivo experiments in mouse***

Male mice (25-35 g), obtained from the animal house of Faculty of Medicine, were kept in controlled environmental conditions (temperature: 23±2 ^o^C; light-dark cycle: 7 a.m. to 7 p.m.) and were divided randomly into groups of seven. All test compounds were dissolved in DMSO and diluted to desire concentration with less than 1% DMSO. 

For the experiment, the test compound (IBMX, milrinone, MCPIP, mc1, mc2, mc5 or mc6) or solvent (control) was injected subcutaneously to mice at 1 mg/kg dosage twice a day (8:00 a.m. and 8:00 p.m.) for 7 days. On day 8, animals were anesthetized with intraperitoneal injection of thiopental (80 mg/kg) and blood samples were obtained from their hearts and then the liver was dissected. Each sample was centrifuged for 5 min and its serum was separated. The serum and the liver of each animal were kept frozen in less than -18 ^o^C for the following measurements.

***In vivo experiments in hyperglycemic rat***

Adult Wistar rats (250-350 g) obtained from the animal house of Faculty of Medicine Mashhad, were kept in controlled environmental conditions (temperature: 23 ± 2 ^o^C; light-dark cycle: 7 a.m. to 7 p.m.), with free access to a standard diet and water. Each rat was fasted for 12-14 hr. The rats were anesthetized with intraperitoneal injection of thiopental (80 mg/kg). Femoral vessels were dissected and the artery and vein were canulated by heparinized catheters. A blood sample was obtained from femoral artery as fasting blood glucose. Then, 0.5 g/kg glucose without (control) or with 1 mg/kg of one of the test compounds was injected via femoral vein. In following, glucose was perfused with a rate of 1.5 g/kg/hr which maintained the hyperglycemic condition. Blood samples were obtained at time intervals of 5, 10, 15, 30, 45, 60, 75 and 90 min via arterial catheter. Each sample centrifuged for 5 min and the serum was separated. The serum and the liver of each animal were frozen in less than -18 ^o^C and kept for the subsequent measurements (11). 

***Liver glycogen storage assay***

To measure liver glycogen storage, 0.5 g of each liver sample was mixed with water and homogenized with a homogenizer, and then 3 ml of 4N HCl was added. To extract the liver glycogen, each sample tube was put in boiling water for 30 min, and then centrifuged for 5 min. In this process, glycogen is hydrolyzed to glucose and released in the medium, 0.5 ml of supernatant was neutralized with 2.5 ml of 1M K_2_HPO_4_ solution. The amount of glucose (mg) in each sample was measured by enzymatic glucose oxidase technique and calculated for one gram liver which multiplied by 0.9 to obtain the amount of glycogen (mg/g liver) (11).

***PDE assay***

**Figure 1:**
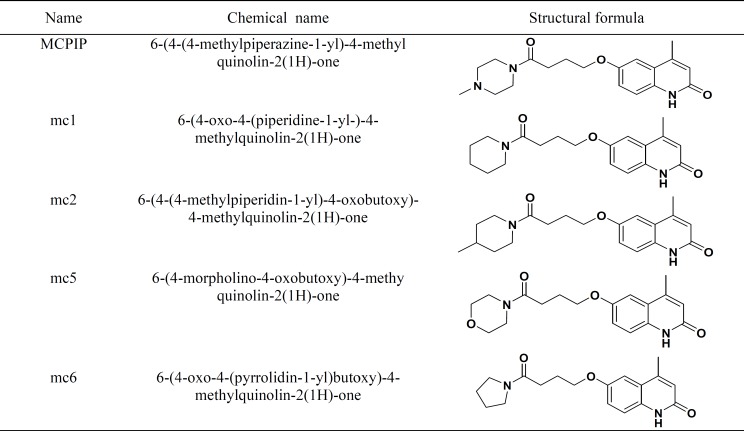
The inhibitory effects of test compounds on PDE3 activity The effects of mentioned test compounds on PDE3B activity. PDE assay is performed by BPS Bioscience Company (**BPS Bioscience** **Inc, San Diego, United** **States). **Each IC_50_ was calculated using Graph Pad Prism software.

PDE3 activity assays of test compounds were performed by BPS Bioscience Company (BPS Bioscience Inc, San Diego, United States) using PDE assay Kit. Fluorescence intensity was measured at an excitation of 485 nm and an emission of 528 nm using a BioTek Synergy^TM^ 2 microplate reader (Figure 1).

***Statistical analysis***

The data were expressed as mean±standard errors of the mean (SEM). In the case of examining more than two groups, one-way analysis of variance (ANOVA) and the Tukey^,,^s* post hoc* test were employed. Differences between means were considered significant if *P*< 0.05. All the obtained data passed a normality test.

**Table1:** Structure of test compounds

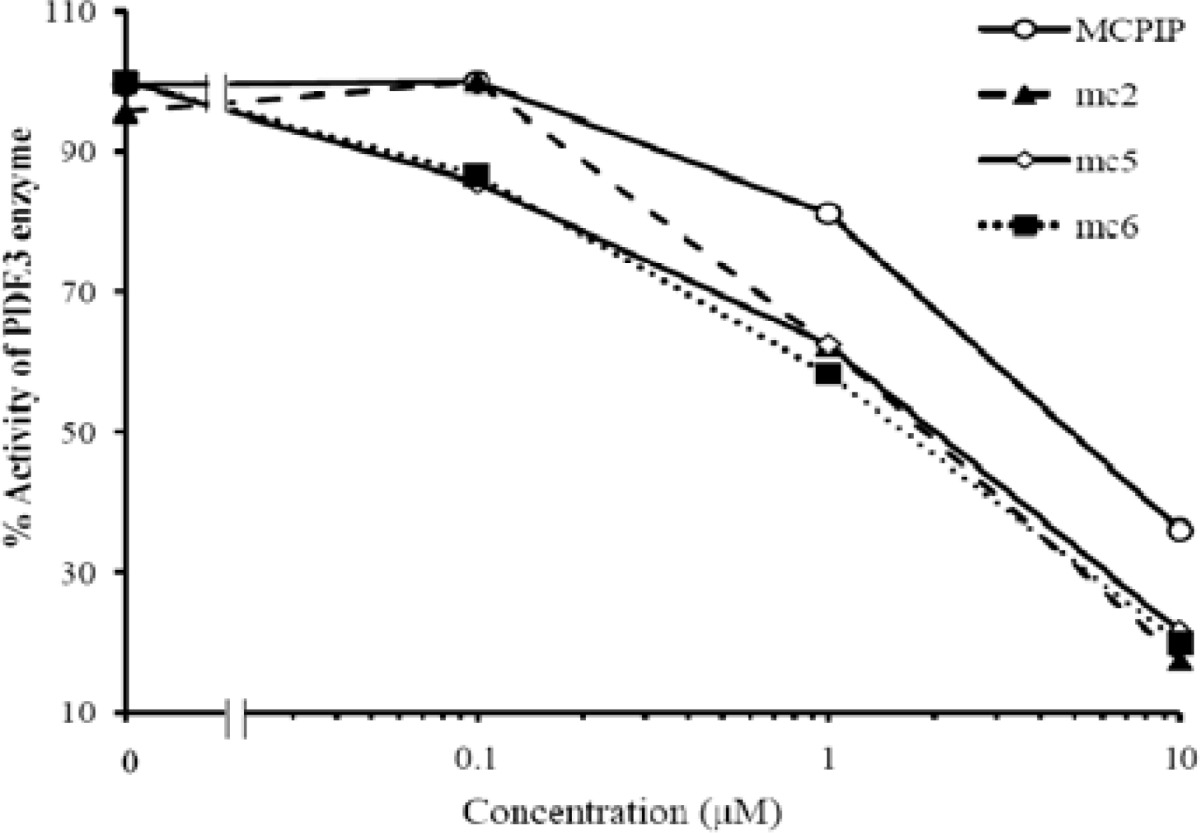

## Results

In this research, we investigated metabolic effects of five new synthetized compounds as selective PDE3 inhibitors, IBMX a non-selective PDE inhibitor and milrinone a selective PDE3 inhibitor on plasma glucose and liver glycogen storage in mice and hyperglycemic rats. 

***Plasma glucose level in mice***

**Figure 2:**
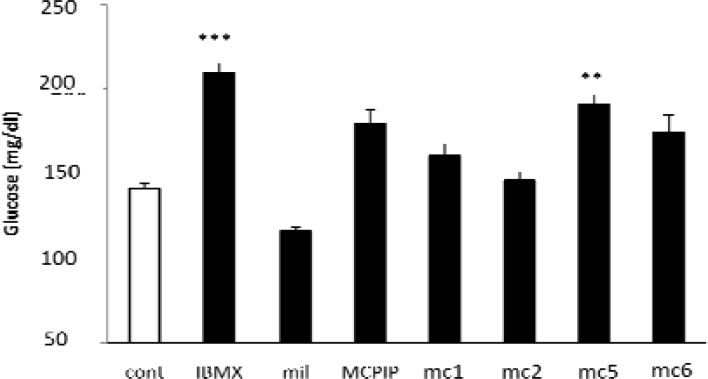
The levels of blood glucose in mice Each mouse was treated for one week by injecting s.c. a test compound (IBMX, Milrinone [mil], MCPIP [PIP], mc1, mc2, mc5 or mc6) or solvent (cont). On day 8, blood samples obtained from heart. Glucose was measured by an enzymatic method. Results are means±SEM from 7 samples. **P< 0.01, ***P< 0.001 indicate significantly difference from control by one-way analysis of variance followed by Turkey's test.

In comparison with the control group, IBMX and mc5 significantly increased plasma glucose (blood glucose, mg/dl, control= 141±3, IBMX= 210±17 *P*< 0.001 and mc5= 191±13 *P*< 0.01) while other test compounds (mc1, mc6, MCPIP and milrinone) did not produce significant effect (control= 141±3, mc1 160±7, mc6 175±9, MCPIP 179±8 and milrinone 116±2 *P*> 0.05) also mc2 did not change plasma glucose (control= 141±3 and mc2= 145±5) (Figure 2). Although it seems that IBMX has the highest efficacy on increasing plasma glucose, its difference with mc5 was not significant.

***Liver glycogen storage level in mice***

**Figure 3:**
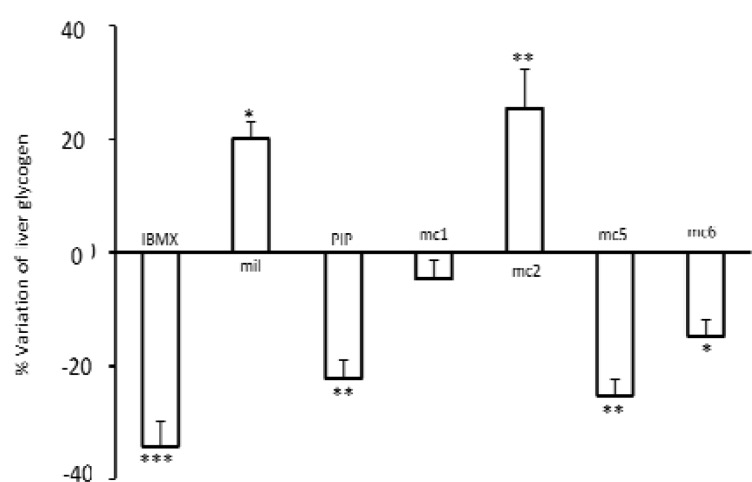
The levels of liver glycogen storage in mice Each mouse was treated for one week by injecting s.c. a test compound (IBMX, Milrinone [mil], MCPIP [PIP], mc1, mc2, mc5 or mc6) or solvent (cont). On day 8, liver was separated for measurement of liver glycogen storage. The amount of glycogen in control was 33±0.7 which its variation considered as zero. The percentage of liver glycogen variation for each test compound is shown as means±SEM from 7 samples. *P< 0.05, **P< 0.01, ***P< 0.001 indicate significantly different from the control by one-way analysis of variance followed by Turkey's test.

Considering that cAMP produces a catabolic effect on glycogen storage, the effects of test compounds on liver glycogen were examined In comparison with the control group (33±0.7) IBMX, a non-selective PDE inhibitor significantly decreased the liver glycogen storage (mg/g, IBMX 22±1.5 *P*< 0.001) and milrinone a selective PDE3 inhibitor significantly increased glycogen storage (control= 33±0.7, milrinone= 40±1 *P*< 0.05). Also mc5, mc6 and MCPIP produced a catabolic effect on liver glycogen (control= 33±0.7, mc5= 25±0.9 and MCPIP= 26±1.1 *P*< 0.01and mc6=28±0.9 *P*< 0.05) (Figure 3). The attitude of effects was comparable to the effect of IBMX. However, mc2, in spite of having a chemical structure similar to these compounds, produced an anabolic effect and significantly elevated liver glycogen storage (control= 33±0.7 and mc2= 42±2.3 *P*< 0.01), while in comparison with the control, mc1 did not change liver glycogen (control= 33±0.7, mc1= 32±1.14 *P*> 0.05) (Figure 3).

***Plasma glucose level in hyperglycemic rats***

In hyperglycemic rat models, loading (0.5 g/kg) and infusion of glucose (1.5 g/kg/hr) augmented blood glucose rapidly and produced a hyperglycemic condition (blood glucose, mg/dl, fasting = 80±4, after 5 min infusion, 265±9). In this situation, IBMX, milrinone, and all the new synthetic compounds reduced significantly the blood glucose (blood glucose, mg/dl, IBMX= 207±9, milrinone= 219±17, MCPIP= 217±2, mc1= 225±3, mc2= 211±5, mc5= 201±4 and mc6= 214±7, comparing with the control, *P*< 0.001). In the control group, blood glucose was decreased gradually reaching to a minimum level within 45 min. Interestingly, this effect was accelerated by all PDE nhibitors but with different efficacies (the amount of blood glucose at minute of 45, mg/dl, control = 203±5, IBMX = 138±7, milrinone = 149±17, MCPIP= 166±4, mc1= 170±4, mc2= 146±5, mc5= 136±7 and mc6= 150±9, in comparison with the control, *P*< 0.001). This effect was potentiated with the combination of milrinone and glybenclamide (129±6 mg/dl). After that, until the end of experiment, in the control group, the blood glucose was increased constantly (blood glucose at minute of 90, mg/dl, control= 240±10). While, during this period, the blood glucose was remained low in the presence of IBMX and increased slightly with selective PDE3 inhibitors (blood glucose at minute of 90, mg/dl, IBMX= 144±6, milrinone= 160±18, MCPIP= 186±2, mc1= 190±8, mc2= 170±3, mc5= 161±6 and mc6= 186±9, in comparison with the control, *P*< 0.001) (Table 2). However, combination of milrinone and glybenclamide reduced blood glucose to a level comparable with fasting condition (mg/dl, fasting= 80±4, milrinone + glybenclamide = 89±2) (Table 2).

**Table 2:** The levels of plasma glucose in hyperglycemic rat

Time (min)	5	10	15	30	45	60	75	90
cont	265±9	244±11	222±6	204±10	203±5	208±8	226±10	240±10
IBMX	207±6***	182±18	154±10	143±9	135±2***	140±3	146±4	144±3***
milrinone	220±12***	198±16	173±11	158±16	146±5***	141±6	146±6	159±10***
MCPIP	217±4***	189±3	180±6	166±7	166±5***	176±3	180±4	186±5***
mc1	225±3***	216±2	187±6	171±4	170±5***	174±8	181±10	190±7***
mc2	211±5***	180±3	166±2	154±3	146±5***	155±7	165±4	170±3***
mc5	201±5***	180±6	166±6	142±5	136±7***	142±4	155±8	161±6***
mc6	214±13***	193±11	180±7	168±11	150±13***	160±1	171±9	186±6***
mil+gly	209±6***	182±20	166±18	150±21	129±6***	87±6	91±2	89±2***

0.5 g/kg glucose accompanies with a test compound (IBMX, Milrinone [mil], Milrinone + Glybenclamide [mil + gly], MCPIP [PIP], mc1, mc2, mc5 or mc6) or solvent (cont) was injected intravenously which was followed by an infusion of 1.5 g/kg/hr glucose. Blood samples were collected in mentioned intervals via arterial catheter. Glucose was measured by an enzymatic method. Each point represents means ± SEM from 7 samples

***P< 0.001, significantly different from the control

***Serum insulin level in hyperglycemic rats***

**Figure 4:**
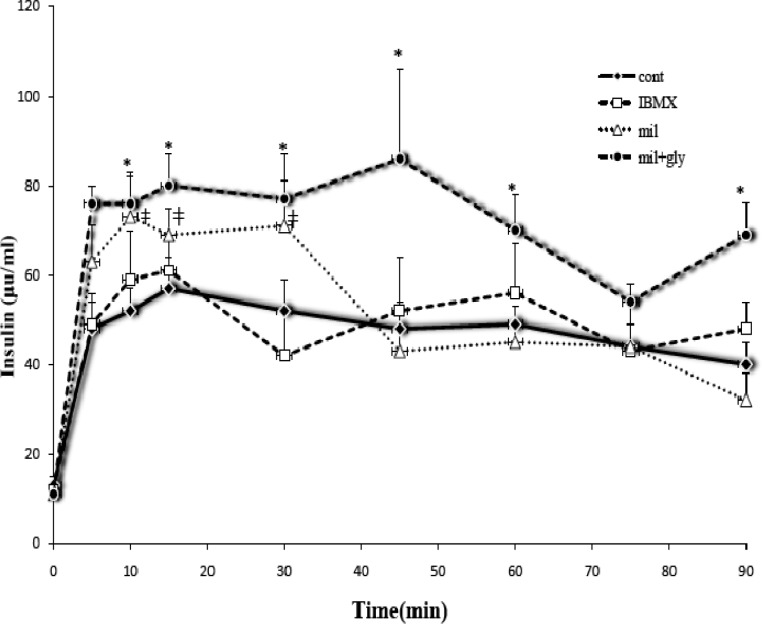
The levels of serum insulin in hyperglycemic rat 0.5 g/kg glucose accompanied with a test compound (IBMX, Milrinone [mil], Milrinone+Glybenclamide [mil+gly]) or solvent (cont) was injected intravenously which was followed by an infusion of 1.5 g/kg/hr glucose. Blood samples were collected in mentioned intervals via arterial catheter. Insulin was measured by RIA method. Each point represents means ± SEM from 7 samples. *) *P*< 0.05 significantly difference between control and combination of milrinone and glybenclamide ( mil+gly) ǂ) *P*< 0.05 significantly difference between control and milrinone (mil)

In the control group, loading and infusion of glucose augmented blood insulin levels rapidly (μIU/ml, fasting = 12±2, after 5 min infusion= 40±5) and remained nearly constant (Figure 4). In comparison with the control, IBMX did not change plasma insulin levels (Figure 4). Milrinone increased glucose-induced insulin secretion initially but after minute of 30, the blood insulin concentrations reduced to level comparable with the control. However, combination of glybenclamide and milrinone increased glucose-induced insulin secretion (*P*< 0.05) through the experiment except at minutes 5 and 75 (Figure 4).

***Glycogen storage level in hyperglycemic rats***

**Figure 5:**
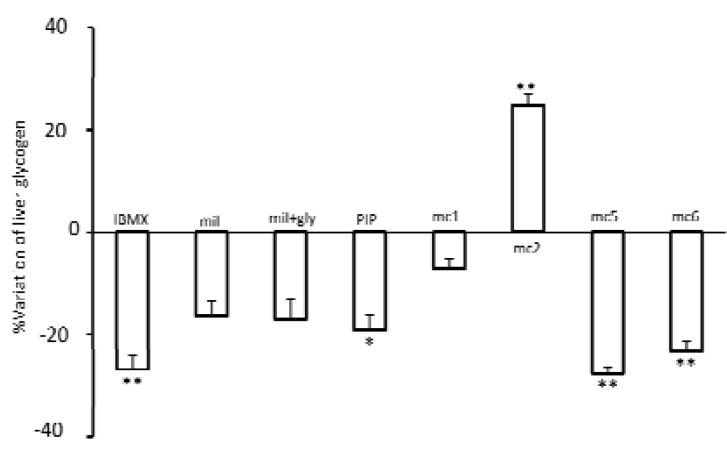
The levels of liver glycogen storage in hyperglycemic rat 0.5 g/kg glucose accompanies with a test compound (IBMX, milrinone [mil], milrinone + glybenclamide [mil + gly], MCPIP [PIP], mc1, mc2, mc5 or mc6) or solvent (cont) was injected intravenously which was followed by an infusion of 1.5 g/kg/hr glucose. Liver was separated at the end of experiment, for measurement of liver glycogen storage. The amount of glycogen in the control was 56±2 which its variation considered as zero. The percentage of liver glycogen variation for each test compound is shown as means±SEM from 7 samples. *P< 0.05, **P< 0.01indicate significantly different from the control by one-way analysis of variance followed by Turkey's test.

We assessed the effect of 90 minutes hyperglycemia on liver glycogen storage in the presence and absence of PDE inhibitors. In comparison with the control group IBMX, mc5, mc6 (*P*< 0.01) and MCPIP (*P*< 0.05) significantly decreased the glycogen storage level (milligram glycogen per gram liver tissue, control= 56±2, IBMX= 40±3, mc5= 41±1.5, mc6= 43±2 *P*< 0.01 and MCPIP= 45±2 *P*< 0.05), while milrinone, milrinone + glybenclamide and mc1 did not significantly decrease glycogen storage (control= 56±2, milrinone= 47±3, milrinone + glybenclamide= 46±4, mc1 =52±2, *P*>0.05). It was remarkable that mc2 produced an anabolic effect and increased liver glycogen storage significantly (control= 56±2, mc2= 70±3, *P*< 0.01) (Figure 5). 

## Discussion

Selective PDE3 inhibitors increase glucose production (1, 18). This is consistent with a key role for PDE3 in insulin-induced antiglycogenolysis in the liver (19). Also inhibition of PDE3B in adipocytes would counteract with the insulin induced antilipolysis, which would increase fatty acid release resulting in insulin resistance (20, 21). Insulin resistance in adipocytes and hepatocytes could further accentuate the negative effects of PDE3 inhibitors in these cells, because insulin utilizes PDE3B to antagonize the effects of catecholamines/cAMP (22, 23).

In the current study, the experimental hyperglycemia condition increased insulin secretion and intravenous glucose loading omitted the effect of glucose on gut increments (24); otherwise, these hormones augment GIIS and potentiate the peripheral insulin effects (25). Therefore, we suggest that this hyperglycemic model is suitable for testing the effects of pharmacological agents on GIIS. In this model, colorimetric blood glucose measuring replaces blood insulin assay which is cheaper, faster, simpler and more stable method.

Methyl-xanthines are potent activators of GIIS and within them IBMX has the highest efficacy and potency (26). However, in this experiment, IBMX, in spite of reducing blood glucose did not change blood insulin levels. It has been shown that, in *in vivo* conditions, the pulsatile pancreatic insulin release characteristics (27) may produce a wide range of variations in plasma insulin concentration within the different animals. This effect hides the effect of IBMX on GIIS. 

Milrinone (a selective PDE3 inhibitor) and glybenclamide (a blocker of K-channels) increase insulin secretion via different mechanism and their combination produces a synergistic effect on GIIS which overcome the pulsative nature of insulin release. Their higher efficacy in lowering of plasma glucose is correlated with the effect of milrinone and glybenclamide on plasma insulin concentrations.

In INS-1 cells, increasing insulin secretion by milrinone is potentiated by glybenclamide (28). 

The level of liver glycogen storage is a balance between glycogenesis and glycogenolysis. Glucose and glucose-6-phosphate stimulate glycogen synthase and decrease glycogenolysis (29). While, activation of cAMP or Ca^2+^ pathways increase glycogenolysis via stimulation of phosphorylase α and inhibition of glycogen synthase (29). In hyperglycemic rat, IBMX significantly decreased liver glycogen storage. Milrinone seems to decrease liver glycogen which was not significant. In combination with glybenclamide, in spite of augmenting plasma insulin levels did not modify the effects of milrinone. This shows the importance of PDE activity for the effect of insulin in liver. This agrees with previous evidences showing non-selective and selective PDE3 inhibitors decrease liver glycogen (4, 7). Among new synthesized compounds mc5, mc6 and MCPIP decreased glycogen storage similar to the effect of IBMX, but mc2 produced an anabolic effect which cannot be related to its inhibitory activity on PDE. 

It has been shown that in skeletal muscle the activity of PDE3 is not important in regulation of cAMP signaling (7). Although inhibition of PDE3 augments GIIS, it did not affect on insulin-induced glucose uptake in skeletal muscle (7) which can explain the effect of test compounds in attenuation of blood glucose. Furthermore, previous investigations indicated that PDE3 inhibitors increase skeletal muscle blood flow which can amplify the uptake of glucose in this tissue (30). Also, vasodilator compounds (e.g. methacholine) increased skeletal muscle glucose uptake in normal subjects while vasoconstrictors (e.g. L-N-monomethylarginine (L-NMMA) an inhibitor of NO synthesis) decrease skeletal muscle glucose uptake (31, 32). 

The long term administration of PDE inhibitor produced differential effect on mouse blood glucose levels and liver glycogen storage. 

The effect of mc2 in increasing liver glycogen storage in rat and mouse is related to its insulinotropic effect with producing weak insulin resistance in both species. However, the differential effects of milrinone on liver glycogen in rat and mouse may suggest that the species-dependent effect of selective PDE3 inhibitors on liver is independent on PDE inhibition. It has been shown that, imazodan is a potent inotropic agent in anesthetized dog while it produces little or no inotropic effect in guinea pig and rat (33). In rat and guinea pig imazodan-sensitive subclass of PDE3 is in a soluble form while in dog, it is in a membrane-form and probably this can play role in different response to imazodan in rat and dog. It has been referred to the presence of species-dependency property for the effects of selective PDE3 inhibitors in heart (33). However, in liver, most of the PDE3 activity is located in particulate and PDE3 inhibition reduces liver glycogen (3). Therefore, the reducing effect of other test compounds on the liver glycogen storage in mouse and hyperglycemic rat may refer to PDE inhibition. The differential effects of test compounds in rat and mouse on liver glycogen storage may be because of their differential indirect mechanisms which need more investigation. IBMX and adenylyl cyclase activators (forskolin) stimulate thyroid hormones secretion that increase glycogenolysis via cAMP-activated pathway (34, 35) and increase endogenous glucose production, hepatic insulin resistance via a sympathetic pathway from the hypothalamic paraventricular nucleus (PVN) to the liver (36). As a result, stimulation of thyroid hormone sensitive neurons in the PVN increases endogenous glucose production by sympathetic projections to the liver (36). 

## Conclusion

Increasing plasma insulin levels by combination of milrinone and glybenclamide confirm that in hyperglycemic rat, the hypoglycemic effect is correlated with increasing insulin secretion. Augmentation of GIIS is obscured by the pulsative characteristic of pancreatic insulin release. Decreasing glycogen storage by IBMX, mc5, mc6 and MCPIP reflects inhibition of liver PDE activity by these compounds which result in insulin resistance in liver. The reasons for ineffectiveness of mc1, anabolic effect of mc2, and differential effects of milrinone is not clear. This may be because of differential inhibitory effect of these compounds on liver PDE activity in *in vivo* condition and may represent a tissue selectivity and/or species-selectivity property.

## References

[1] Degerman EE, Manganiello V, Ahren B (2004). Milrinone efficiently potentiates insulin secretion induced by orally but not intravenously administered glucose in C57BL6J mice. Eur J Pharmacol.

[2] Shafiee-Nick R, Pyne NJ, Furman BL (1995). Effects of type-selective phosphodiesterase inhibitors on glucose-induced insulin secretion and islet phosphodiesterase activity. Br J Pharmacol.

[3] Harndahl L, Jing X-J, Iverson R, Degerman E, Ahrén B, Manganiello VC (2002). Important role of phosphodiesrerase3B for the stimulatory action of cAMP on pancreatic β-cell exocytosis and release of insulin. J Biol Chem.

[4] Zumuda -TrzebiatowskaE, Oknianska A, Manganiello V (2006). Role of DE3B in insulin-induced glucose uptake, GLU-T4 translocation and lipogenesis in primary rat adipocytes. Cell Signal.

[5] Maurice DH, Palmer D, Tilley D, Dunkerley HA, Netherton SJ, Raymond DR (2003). Cyclic nucleotide phosphodiesterase activity, expression and targeting in cells of the cardiovascular system. Mol Pharmacol.

[6] Pyne NJ, Furman BL (2003). Cyclic nucleotide phosphodiesterases in pancreatic islets. Diabetologia.

[7] Cheung P, Yang G, Boden G (2003). Milrinone a selective phosphodiesterase3 inhibitor stimulates lipolysis, endogenous glucose production and insulin secretion. Metabolism.

[8] Parizadeh SMR, Shafiee NikR, Zahraei M (2001). Comparison between the insulinotropic effects of milrinone and amrinone, selective PDE3 inhibitors in in-vitro and in-vivo conditions. Iran J Basic Med Sci.

[9] Mansouri MT, Shafiee-Nick R, Parsaee H, Seyedi SM, Saberi MR, Sadeghian H (2008). Inotropic and chronotropic effects of 6-hydroxy-4-methylquinolinone-2 (1H)-one derivatives in isolated rat atria. Iran Biomed J.

[10] Sadeghian H, Seyedi M, Saberi MR, Nick RS, Hosseini A, Bakavoli M (2008). Design, synthesis and pharmacological evaluation of 6-hydroxy-4-methylquinolin-2 (1H)-one derivatives as inotropic agents. J Enzyme Inhib Med Chem.

[11] Pour AliBehzadN, Shafiee NickR, Parizadeh SMR (2007). Anti-hyperglycemic effects of cyclic nucleotides phosphodiesterases (PDEs) in rat. Med J Tabriz Univ Med Sci.

[12] Waddleton D, Wub W, Feng Y, Thompson C, Wu M, Zhou YP (2008). Phosphodiesterase 3 and 4 comprise the major cAMP metabolizing enzymes responsible for insulin secretion in INS-1 (832/13) cells and rat islets. Biochem Pharmacol.

[13] Ahmad M, Abdel-Wahab YHA, Tate R, Flatt PR, Pyne NJ, Furman BL (2000). Effect of type-selective inhibitors on cyclic nucleotide phosphodiesterase activity and insulin secretion in the clonal insulin secreting cell line BRIN-BD11. Br J Pharmacol.

[14] EL-Metwally M, Shafiee-Nick R, Pyne NJ, Furman BL (1997). Effects of Org 9935, a selective type III phosphodiesterase inhibitor and Org 30029 a mixed type III/IV phosphodiesterase inhibitor on glucose-induced insulin secretion in vivo and in vitro. Eur J Pharmacol.

[15] Yang G, Li L (2003). In vivo effects of phosphodiesterase III inhibitors on glucose metabolism and insulin sensitivity. J Chin Med Assoc.

[16] Parker JC, VanVolkenburg MA, Nardone NA, Hargrove DM, Andrews KM (1997). Modulation of insulin secretion and glycemia by selective inhibition of cyclic AMP phosphodiesterase III. Biochem Biophys Res Commun.

[17] Furman BL, Pyne NJ (1990). Islet phosphodiesterase isoenzymes and insulin secretion. Diabetic Med.

[18] Zmuda-Trzebiatowskaa E, Oknianskaa A, Manganiellob V, Degerman E (2006). Role of PDE3B in insulin-induced glucose uptake, GLUT-4 translocation`` and lipogenesis in primary rat adipocytes. Cell Signal.

[19] Beebe SJ, Redmon JB, Blackmore PF, Corbin JD (1985). Discriminative insulin antagonism of stimulatory effects of various cAMP analogs on adipocyte lipolysis and hepatocytes glycogenolysis. J Biol Chem.

[20] Arner P (2002). Insulin resistance in type 2 diabetes: role of fatty acids. Diabetes Metab Res Rev.

[21] Boden G (2003). Effects of free fatty acids (FFA) on glucose metabolism: significance for insulin resistance and type 2 diabetes. Exp Clin Endocrine Diabetes.

[22] Eriksson H, Ridderstrale M, Degerman E, Ekholm D, Smith CJ, Manganiello VC (1995). Evidence for the key role of the adipocyte cGMP-inhibited cAMP phosphodiesterase in the antilipolytic action of insulin. Biochem Biophys Acta.

[23] Hagstrfm-Toft E, Bolinder J, Eriksson S, Arner P (1995). Role of phosphodiesterase III in the antilipolytic effect of insulin in vivo. Diabetes.

[24] Zhao AZ, Karin A, Beavo JA (1998). Leptin inhibits insulin secretion by activation of PDE3B. J Clin Invest.

[25] Yamada S, Komatsu M, Sato Y, Yamauchi K, Kojima I (2002). Time dependent stimulation of insulin exocytosis by 3, 5 cyclic adenosine monophosphate in the rat islet β-cells. Endocrinology.

[26] Doyle M, Egan J (2006). Pharmacological agents that directly modulate insulin secretion. Pharmacol Rev.

[27] Porksen N, Hollingdal M, Juhl C, Butler P, Veldhuis JD, Schmitz O (2002). Pulsatile insulin secretion: detection, egulation, and role in diabetes. Diabetes.

[28] Parker JC, Volkenburg MA, Gao Feng (2004). Synergistic effect on insulin secretion from INS-1 cells of a sulfonylurea and a phosphodiesterase 3 inhibitor. Life Sci.

[29] Bollen M, Keppens S, Stalmans W (1998). Specific features of glycogen metabolism in the liver. Biochem J.

[30] Fong M, Yoshitake M, Kambayashi J, Liu Y (2010). Cilostazol increases tissue blood flow in contracting rabbit gastrocnemius muscle. Circ J.

[31] Baron AD, Steinberg H, Brechtel G, Johnson A (1994). Skeletal muscle blood flow independently modulates insulin-mediated glucose uptake. Am J Physiol.

[32] Baron AD, Steinberg HO, Chaker H, Irsula O, Brechtel G, Keech C (1995). Insulin-mediated skeletal muscle vasodilatation contributes to both insulin sensitivity and responsiveness in lean humans. J Clin Invest.

[33] Weishaar RE, Kobylarz-Singer DC, Steffen RP, Kaplan HR (1987). Subclasses of cyclic AMP-specific phosphodiesterase in left ventricular muscle and their involvement in regulating myocardial contractility. Circ Res.

[34] Laurberg P (1984). Forskolin stimulation of thyroid secretion of T4 and T3. FEBS.

[35] Schery MP, Brown BL, Ekins RP (1978). Studies on the role of calcium and cyclic nucleotide in the control of TSH secretion. Mol Cell Endocrine.

[36] Klieverik LP, Janssena SF, Riel A, Foppen E, Bisschop PH, Serlie MJ (2009). Thyroid hormone modulates glucose production via a sympathetic pathway from the hypothalamic paraventricular nucleus to the liver. Proc Natl Acad Sci U S A.

